# Tacrolimus-Associated Dilated Cardiomyopathy in Adult Patient After Orthotopic Liver Transplant

**DOI:** 10.1177/2324709617706087

**Published:** 2017-04-27

**Authors:** Jennifer McLeod, Stephanie Wu, Luanda Grazette, Anna Sarcon

**Affiliations:** 1 University of Southern California, Los Angeles, CA, USA

**Keywords:** dilated cardiomyopathy, tacrolimus

## Abstract

This report presents a case of tacrolimus cardiotoxicity in an adult patient who received tacrolimus immunosuppression for orthotopic liver transplant (OLT). Tacrolimus-associated cardiotoxicity has been described in the literature, however this is the first case to document the development of a dilated cardiomyopathy in a patient shortly after initiating tacrolimus therapy post transplant. With the growing use of tacrolimus in transplant medicine, this case report expands the literature of tacrolimus cardiotoxicity and can aid clinicians in the evaluation and management of patients exposed to this form of immunosuppression.

## Introduction

Tacrolimus is a potent immunosuppressive agent that has improved the success of solid organ transplant. In comparison to cyclosporine, tacrolimus has been shown to be superior in liver transplant for graft and patient survival, and it is associated with fewer rejection episodes.^1^ Upon entering T cells, tacrolimus forms a complex with FK-binding proteins to inhibit calcineurin, leading to inactivation of T lymphocytes. However, the calcineurin binding protein can also be found in nervous, skeletal, and cardiac tissue, allowing for the possibility of adverse systemic effects. It is suspected that calcineurin inhibition can alter sympathetic activation or potentially influence calcium release channels, contributing to the development of neurotoxicity, nephrotoxicity, and cardiac toxicity.^2^ Adverse renal, central nervous system, and vascular effects have been previously documented. Nonetheless, there is little literature regarding toxic effects on the heart.^3-5^ The available case reports on this topic have documented incidences of a hypertrophic cardiomyopathy in pediatric and adult human transplant recipients while on tacrolimus.^3,4,6,7^ However, the phenotype documented in these cases involve septal or free wall hypertrophy with or without ventricular outflow track obstruction. Our case is the first to describe a tacrolimus-associated dilated cardiomyopathy with severely reduced systolic function in an orthotopic liver transplant (OLT) patient.

## Case

We report the case of a 59-year-old Hispanic male with Child Class C cirrhosis, due to alcohol abuse, who received an OLT (MELD 43 at time of transplant). Pretransplant echocardiogram showed left ventricular ejection fraction (LVEF) of 72% with normal left ventricular thickness. Pretransplant coronary angiogram showed normal coronary arteries. Subsequent echocardiograms after liver transplant continued to show normal LVEF. By posttransplant day 2, the patient was started on oral tacrolimus 1 mg every 12 hours and was titrated to 4 mg every 12 hours within 48 hours (0.1-0.15 mg/kg/day dosing). The patient was also started on methylprednisolone and mycophenolate mofetil for immunosuppression. During posttransplant monitoring, the 12-hour whole blood tacrolimus levels, analyzed through the ELISA method, remained below 15 mg/mL (per the 1995 Consensus Document of tacrolimus therapeutic monitoring, the target whole blood tacrolimus trough concentrations are 5-20 mg/mL early posttransplant^8^). The patient’s renal function remained stable, with a serum creatinine at his baseline of 0.8 to 0.9 mg/dL throughout the hospitalization. Once the patient remained stable, he was discharged on tacrolimus 4 mg every 12 hours.

After discharge, the patient had complaints of persistent pain secondary to lumbar and thoracic compression fractures; he was shortly readmitted for a scheduled thoracic kyphoplasty. A preoperative echocardiogram (posttransplant day 63) showed significantly reduced LVEF of 35% with hypokinesis of anterior, anteroseptal, and inferoseptal walls and akinesis of the mid inferior and inferolateral walls, and severe asymmetric left ventricular hypertrophy. Cardiology was consulted and patient was started on a beta-blocker and angiotensin-converting enzyme inhibitor, as well as a diuretic given findings of pulmonary edema. Whole blood tacrolimus levels remained 7 to 10 ng/mL throughout this hospitalization. The patient was then discharged a few days later without changes made to his immunosuppressive regimen.

**Figure 1.**
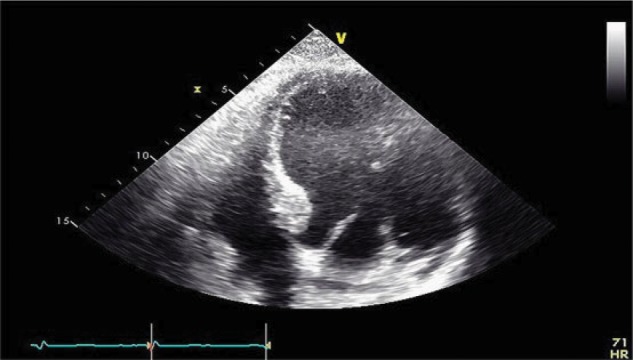
Four-chamber view on 2D transthoracic echocardiogram showing aneurysmal dilation of the left ventricular mid/apical segments, in end-diastole of cardiac cycle.

**Figure 2.**
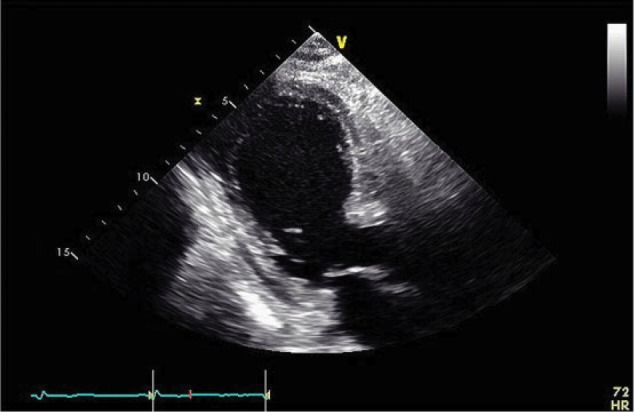
Parasternal long view on 2D transthoracic echocardiogram showing moderate ventricular septum and free wall thickness.

**Figure 3.**
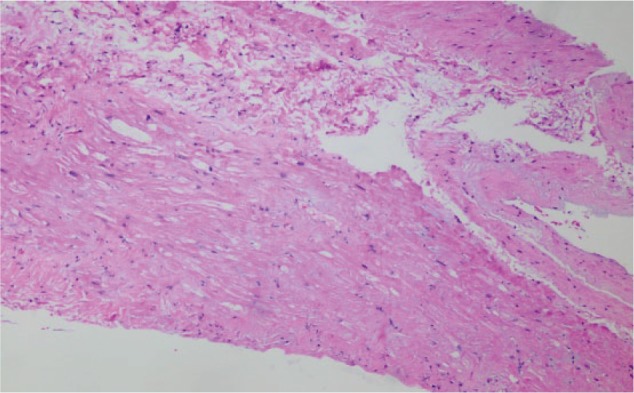
Endomyocardial biopsy with hematoxylin-eosin staining, showing area of focal mild fibrosis. No evidence of eosinophil or giant cell infiltration and no viral type changes identified. Immunofluorescence staining was negative for immune complex deposition and negative Congo red staining (not shown).

Approximately 3 months after transplant, the patient was readmitted for acute encephalopathy and tacrolimus was discontinued given mental status changes (whole blood tacrolimus level 6.7 at the time of admission). Repeat echocardiogram showed worsening LVEF (20%) and aneurysmal dilatation and dyskinesis of mid/apical segments of LV, concerning for large territory infarction or stress-induced cardiomyopathy (Figures 1 and 2). Thus, the patient underwent a repeat coronary angiogram showing normal coronary arteries as well as endomyocardial biopsy, which showed focal fibrosis (Figure 3). Infectious workup including cytomegalovirus, Epstein-Barr virus, hepatitis B and C, and varicella were negative. After tacrolimus was held for 4 days, the patient’s mental status returned to baseline. The patient was subsequently discharged on cyclosporine, given concern that tacrolimus may have caused his acute cardiomyopathy and encephalopathy.

## Discussion

The cardiotoxic effects of tacrolimus in patients requiring immunosuppression after transplant has been previously described in the literature. However, this is the first case to document progression to a dilated cardiomyopathy in a tacrolimus-treated adult OLT recipient.

Earlier case reports and prospective studies have described the development of a hypertrophic cardiomyopathy in patients being treated with tacrolimus. Roberts et al found septal hypertrophy among the autopsy hearts of adult and pediatric OLT patients; these patients were identified to not have risk factors for cardiac disease prior to transplant.^7^ Atkison et al described a pattern of concentric hypertrophy in pediatric patients on imaging, which resolved or showed improvement after tacrolimus was discontinued.^4^ Similarly, Uemoto et al presented an abstract at the 15th Annual Meeting of the American Society of Transplant Physicians, describing a cohort of pediatric liver transplant recipients on tacrolimus therapy who developed reversible cardiac wall thickening within the left ventricle and interventricular septum by 1 to 2 weeks posttransplant.^5^

The patient from our case report described above had a similar development of cardiac hypertrophy shortly after the introduction of tacrolimus, particularly among the intraventricular septum. However, our case is starkly unique in that the patient rapidly progressed to a dilated cardiomyopathy, which remained unchanged after discontinuation of the drug. Given the brief time period between the initiation of immunosuppression and the abrupt changes in cardiac function, it is plausible that tacrolimus is the culprit in our patient. This case is the first to document a dilated cardiomyopathy attributable to tacrolimus therapy in an adult human patient after OLT.

The mechanism of tacrolimus-associated cardiomyopathy in OLT is unclear.^9^ Proposed mechanisms include coronary artery arteritis, cardiac tissue calcification, and tacrolimus-induced hypertension from sympathetic activation.^6^ While the great majority of patients on tacrolimus therapy do not develop cardiotoxicity, this case demonstrated a phenotype never before described, and that may not be readily reversible, as documented in prior cases.^10,11^ With the growing use of tacrolimus in transplant medicine, more attention is needed to understand its full range of adverse effects. The described case contributes to the literature of tacrolimus cardiotoxicity and brings attention to the need for careful evaluation of patients exposed to this form of immunosuppression.
